# Diffusion Tensor Imaging (DTI) as a Non‐Invasive Tool for Assessing Pediatric Kidney Transplants: A Feasibility Study

**DOI:** 10.1111/petr.70119

**Published:** 2025-06-11

**Authors:** Suraj D. Serai, Tatiana Morales, Daniel Vossough, Sandra Amaral, Hansel J. Otero, Bernarda Viteri Baquerizo

**Affiliations:** ^1^ Department of Radiology Children's Hospital of Philadelphia Philadelphia Pennsylvania USA; ^2^ Perelman School of Medicine at the University of Pennsylvania Philadelphia Pennsylvania USA; ^3^ Department of Pediatrics, Division of Nephrology Children's Hospital of Philadelphia Philadelphia Pennsylvania USA; ^4^ Department of Biostatistics, Epidemiology, and Informatics Perelman School of Medicine at the University of Pennsylvania Philadelphia Pennsylvania USA

**Keywords:** DTI, kidney transplant, pediatric

## Abstract

**Background:**

Pediatric kidney transplant recipients require periodic biopsy for active surveillance to prolong allograft half‐life, and non‐invasive MR imaging markers are needed but understudied. Here we aimed to determine the feasibility of MR diffusion tensor imaging (DTI) on pediatric kidney transplant recipients, compare transplanted kidneys DTI values to healthy controls, and correlate DTI values with allograft histopathology.

**Methods:**

Single‐center prospective study of pediatric (< 18 years of age) kidney transplant recipients undergoing biopsies and healthy controls between February 2020 and October 2023. MRI DTI‐derived metrics (fractional anisotropy [FA] and track length) of the kidney cortex were obtained for all participants. Transplant recipients versus controls, rejection versus non‐rejection, and high chronic allograft damage index (CADI) versus low were compared using two‐sample t‐test or Wilcoxon rank‐sum test.

**Results:**

Fifteen transplant recipients (4F/11M, median 16 [IQR 13–18] years old) and 15 healthy controls (9F/6M, median 15 [IQR 12–22] years old, 30 kidney units) were evaluated. DTI was technically appropriate in all cases. Smaller FA values and longer track length were found in allografts (FA in allografts (median [IQR]: 0.25 [0.25–0.28]) vs. controls (0.28 [0.27–0.33], *p* = 0.003) and track length in allografts (mean: 19.36 ± 5.21) vs. controls (12.80 ± 3.34, *p*‐value < 0.001). FA and track length between allografts with and without rejection, and/or with high vs. low CADI score were not significantly different.

**Conclusion:**

DTI in pediatric kidney transplants is feasible and showed differences in FA and track length values when compared to controls. However, in our limited dataset, DTI did not find differences within the allograft group.

## Graphical abstract

Our study demonstrates the feasibility of using diffusion tensor imaging (DTI) to evaluate pediatric kidney transplants. Significant differences in fractional anisotropy (FA) and track length were observed between transplanted and healthy kidneys, reflecting altered microstructural organization. While DTI metrics did not distinguish allografts by rejection status or chronic damage index, the findings support DTI's potential as a non‐invasive biomarker for assessing kidney allograft health. Further research with larger cohorts is needed to validate these results and explore the clinical utility of DTI in pediatric kidney transplant management.

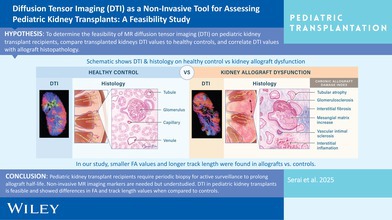

## 1 Introduction

Kidney transplantation is the gold standard treatment for end‐stage kidney disease (ESKD), offering superior survival and quality of life compared to dialysis. However, patients undergoing kidney transplantation have unacceptably poor survival rates, especially children, whose lifespan is from 20 to 25 years shorter than that of other children [1], often requiring multiple kidney allografts over their lifetime [2].

Kidney allograft injury results from acute cellular or antibody‐mediated rejection, ischemia reperfusion injury, vascular complications, viral infections, chronic allograft nephropathy, among other insults [3] that accumulate over time and lead to the progression of chronic kidney disease (CKD) and allograft loss. The current gold standard to identify kidney allograft injury is biopsy, despite its limitations and complications, including fistula formation [4, 5, 6]. Serum creatinine and its estimation of glomerular filtration rate (eGFR) have been the primary non‐invasive biomarkers for the detection of allografts at risk of failure; however, they are limited by their low sensitivity and specificity [7, 8]. Therefore, there is much interest in establishing robust non‐invasive quantitative imaging biomarkers for the detection of kidney allograft injury, minimizing the number of required biopsies, and improving the long‐term survival of each allograft [9, 10, 11, 12].

**FIGURE 1:**
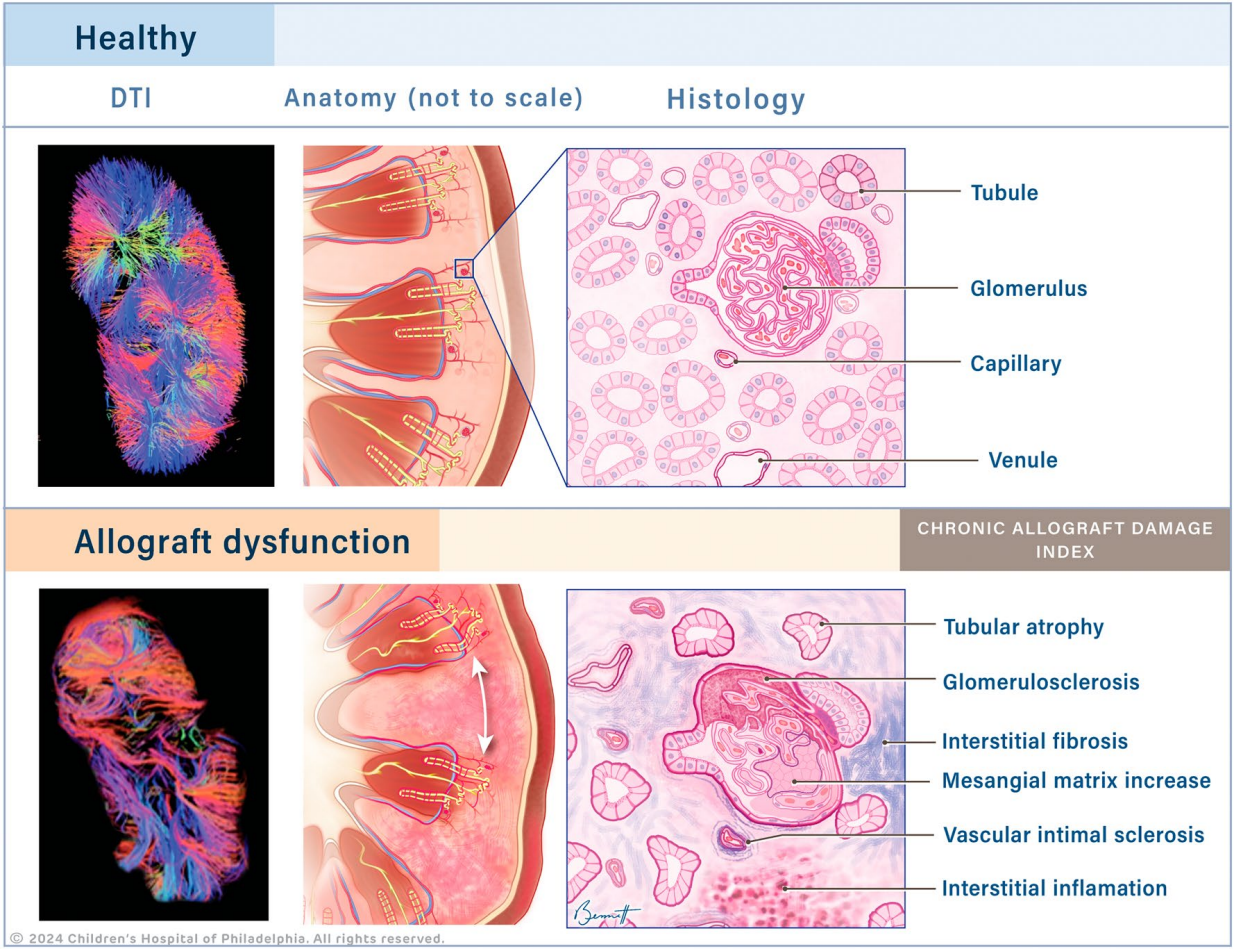
Schematic showing DTI vs. histology on healthy control vs. kidney allograft dysfunction.

Nephrons in healthy kidneys are well organized within the parenchyma and follow a radial arrangement [13, 14]. However, in patients with CKD, as the disease progresses, there is displacement of coherent kidney tubule orientation [15]. These microstructural alterations can be quantified using magnetic resonance imaging (MRI)‐based diffusion‐based imaging (Figure 1) [14, 16, 17].

**FIGURE 2:**
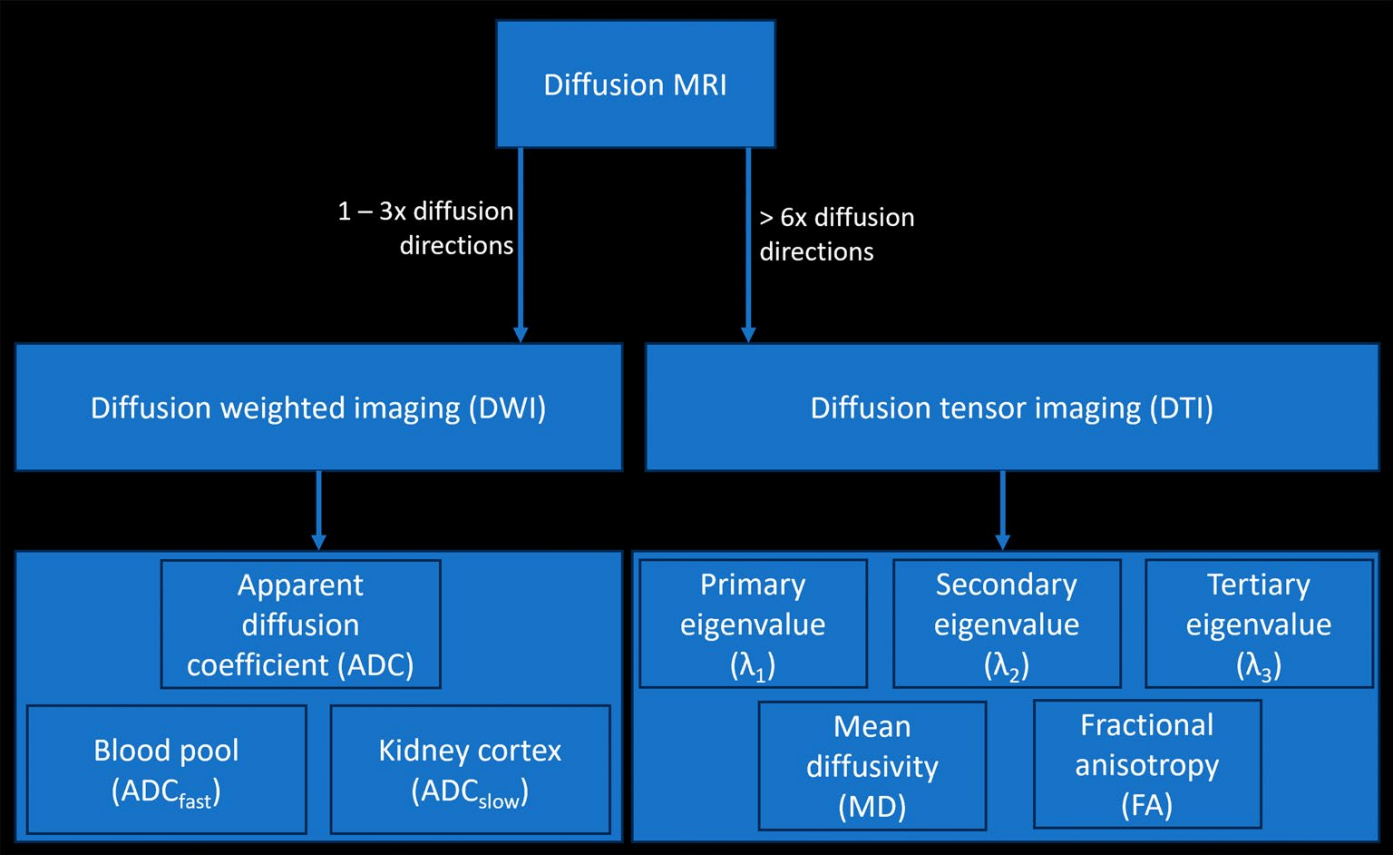
Diffusion terminology. Low and high diffusion‐weighted images are acquired, then used to reconstruct ADC or DTI maps (MD and FA maps). Primary, secondary, and tertiary eigenvalues are calculated from the diffusion tensor and used to derive commonly used metrics of diffusivity and microstructure in the kidney. ADC, apparent diffusion coefficient; DTI, diffusion tensor imaging; DWI, diffusion weighted imaging; FA, fractional anisotropy; MD, mean diffusivity.

Diffusion tensor imaging (DTI) is based on the application of diffusion gradients in different directions in space, enabling the evaluation of the movement of water molecules in three dimensions and whether there is a dominant direction to diffusion restriction [18, 19, 20]. Hence, it can be used to quantitatively assess the microstructures and morphologies of different tissues in the body, including the kidney (Figure 2). DTI provides quantitative parameters, including ADC or also called mean diffusivity (MD), fractional anisotropy (FA), track length, and track volume, and also allows the generation of tractography‐based maps [16, 21]. ADC reflects the magnitude of water's mobility, while FA is determined by its directionality, reflecting the dominance of one particular water movement direction in a voxel. The orientation and length of fiber tracks may be measured according to the dominant direction of water movement in each voxel [16]. Fiber tracking is based on the dominant direction of water movement in each voxel.

DTI‐MRI has been utilized for over a decade to measure tissue damage in other organs (such as the brain), but its application in kidney disease is more recent [16, 17, 22]. The ability of DTI to quantify the directional nature of water movement makes it ideally suited for analyzing highly structured organs such as the kidney [23]. Additionally, DTI does not require the administration of contrast agents, making it suitable for subjects with kidney dysfunction, for which the use of such agents is typically contraindicated.

The purpose of our study is to evaluate the feasibility of DTI on pediatric kidney transplant recipients, determine if transplanted kidneys have differing quantitative values when compared to healthy controls, and evaluate the correlation of DTI quantitative parameters with histopathologic findings.

## 2 Methods

### 2.1 Study Design and Study Sample

This is a prospective NIH‐funded, single‐center feasibility pilot study of “Imaging Modalities in Pediatric Assessments of Kidney Transplant” (IMPAKT). As part of IMPAKT, pediatric kidney transplant recipients who are 6 years and older and have a “for‐cause” or “surveillance” biopsy (at 6, 12, or 24‐months post‐transplantation) as part of their routine clinical care, are consented to undergo MRI and advanced ultrasound imaging within 48 h *prior* to biopsy. Imaging studies were planned to be done during the patient's clinical visit to the hospital. For quantitative comparison purposes, healthy pediatric volunteers without any history of kidney disease and BMI between 5th and 85th percentile are recruited from the institutional pool of volunteers through the recruitment enhancement core of the Research Institute at Children's Hospital of Philadelphia (CHOP). The study was approved by the ethics committee of CHOP. Informed consent and, when applicable, assent was obtained from all participants. The general contraindications for MRI were applied to the screening process. All participants underwent non‐sedated and non‐contrast MRI. Serum creatinine and cystatin‐C, markers of kidney function, were collected no more than 36 h before or after imaging studies for all participants. eGFR was calculated based on the U25‐eGFR serum creatinine and cystatin‐C combined equation, for which we will refer to as U25‐Average eGFR [24]. Urinalyses are also collected as part of IMPAKT within 1 week of imaging studies. Clinical data on immunosuppressive therapy at the time of biopsy, as well as any transplant complications, were extracted from the electronic medical record.

### 2.2 Pathology

As part of the IMPAKT study, routine pathological sections and immunohistochemistry‐stained sections were independently evaluated by an experienced pediatric pathologist (TB). The Banff classification was updated in 2017 to improve the diagnosis and grading of rejection in kidney allografts. Compared to the 2013 version, which evaluated cortical and medullary inflammation separately, the 2017 revision introduced a combined inflammation score those accounts for both compartments [25]. It also refined the definitions of borderline rejection and chronic active T‐cell‐mediated rejection. These changes aimed to better capture the overall inflammatory burden in the allograft. The Banff 2017 criteria were used to classify rejection and allograft injury [26]. Rejection was defined as “antibody‐mediated rejection” (acute and chronic) and/or “T‐cell mediated rejection” (acute and chronic), including “borderline T‐cell‐mediated rejection” Banff 2017 category 2, 4, and 3, respectively. To assess chronic damage of allograft tissue, the chronic allograft damage index (CADI), Figure 1, was calculated based on the sum of 0 to 3 score (based on severity “0” = none to “3” = severe) of interstitial inflammation (i), interstitial fibrosis (ci), tubular atrophy (ct), increased basement membrane matrix (mm), glomerular sclerosis (g) and vascular intimal sclerosis (cv) [27]. With a maximum score of 18, increased risk of allograft loss and worse outcomes have been associated with CADI score 2+ [27, 28, 29, 30].

### 2.3 MR Imaging

**FIGURE 3:**
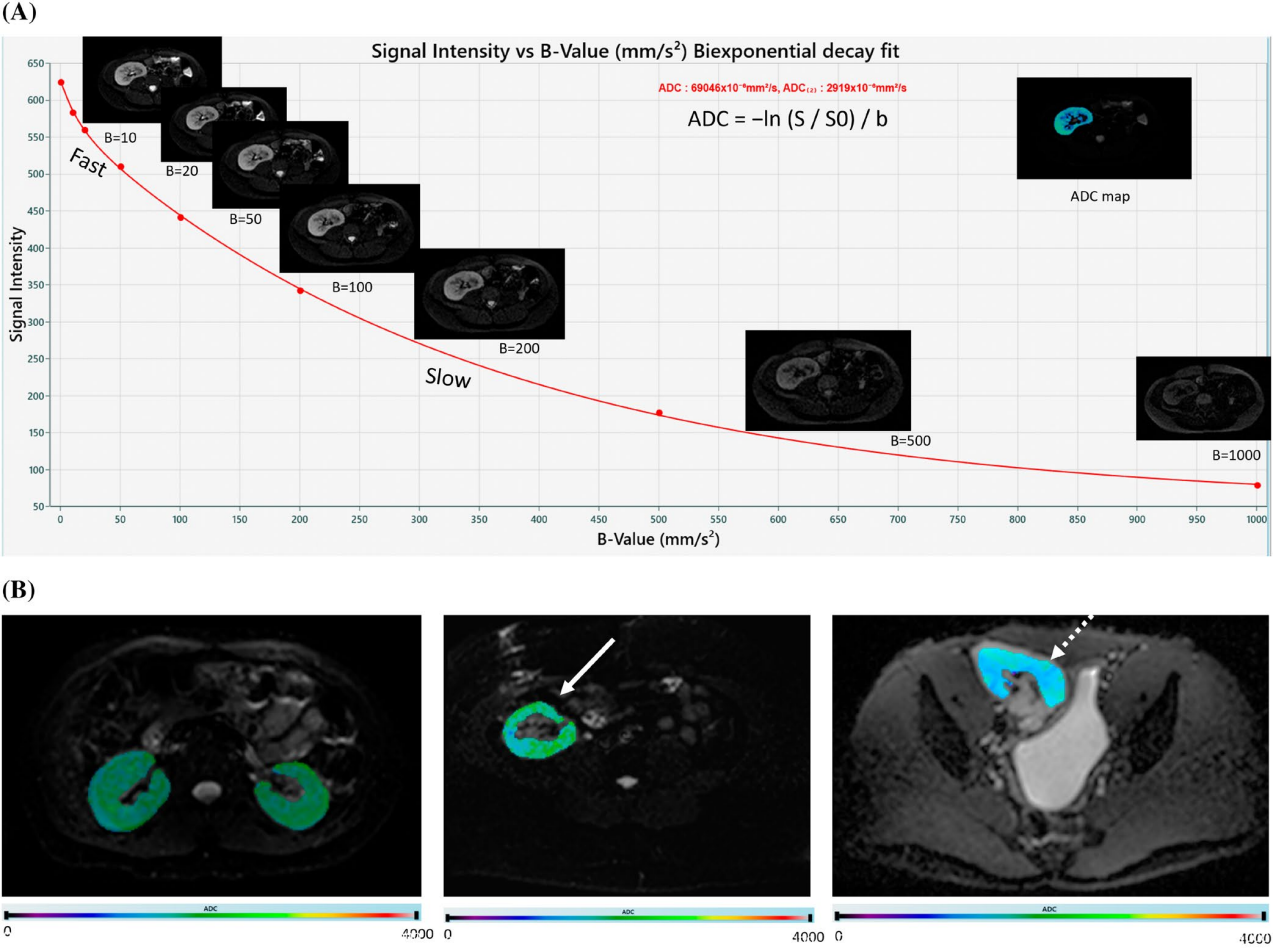
Representative DWI. (A) b‐value images showing post‐processing fitting to generate ADC maps, (slow diffusion or pure‐diffusion component: Brownian motion of water molecules; fast diffusion or pseudo‐diffusion component: Microcirculation of intravascular molecules in the micro‐capillaries), (B) diffusion‐generated ADC maps overlaid on B0 image for healthy control with native kidneys as compared to a patient with stable transplanted kidney allograft and another patient with rejected transplanted kidney allograft. Lower ADC value is observed on the patient with rejected allograft.

**FIGURE 4:**
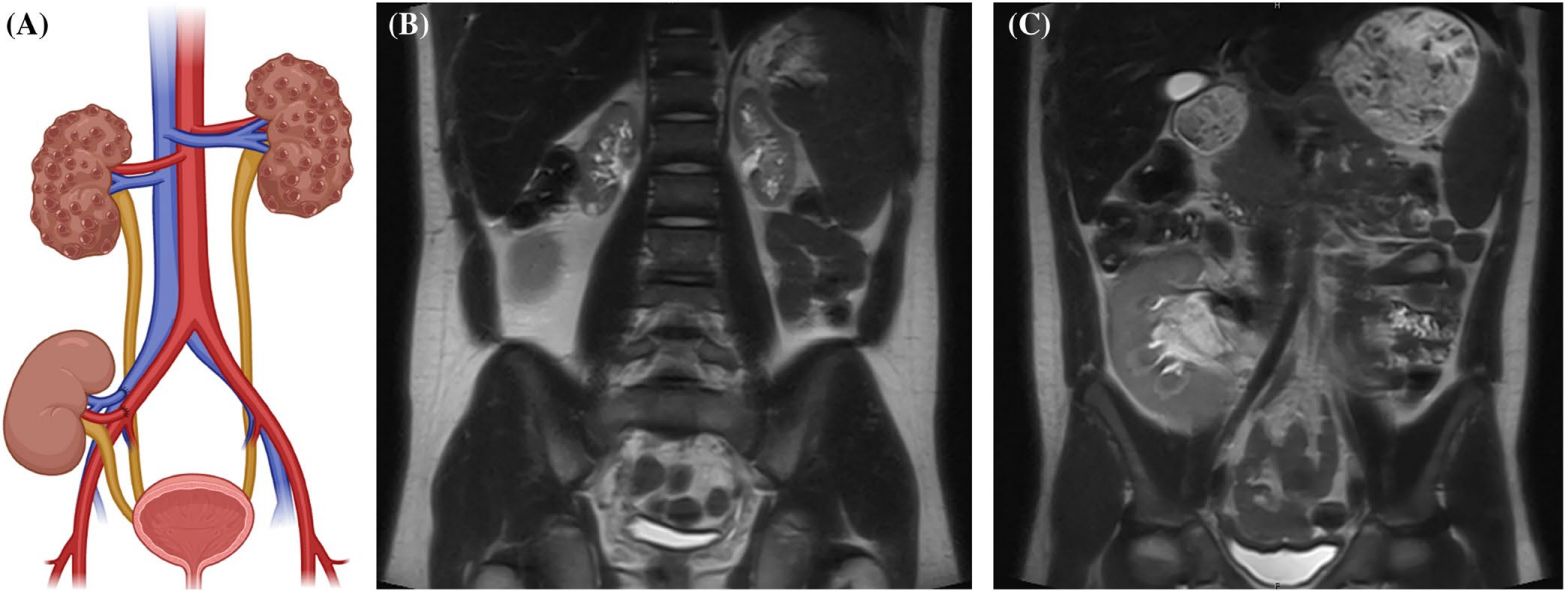
Fourteen‐year‐old boy with kidney transplant. (A) Schematic image, (B) coronal T2W image showing the atrophic bilateral native kidneys and (C) same coronal T2W series with the anterior slice showing transplanted kidney.

**FIGURE 5:**
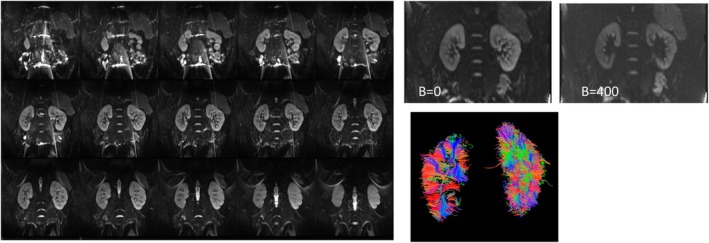
Representative DTI of an 18‐year‐old control female subject showing the kidney slices coverage, B0 image, B400 image of a 20‐direction DTI acquired on a Siemens 3 T MRI. An inset shows the post‐processed DTI tracks.

A series of coronal single shot image series is first acquired to identify the location of the allograft (typically placed extra‐peritoneally in the right or left lower quadrant) **(**Figures 3 and 4 **)**. DTI is typically obtained by acquiring a set of spin‐echo echo‐planar imaging (EPI) sequences with different diffusion directions and 2 b‐values [14, 31, 32]. As part of IMPAKT, DTI‐MRI of the kidney is obtained in an oblique coronal plane using a fat‐suppressed spin‐echo EPI sequence on a 3 T MR scanner (Siemens Healthineers, PA, USA). A slice thickness of 3 mm, a matrix size of 128 × 128, and a bandwidth of ~1776 Hz/pixel is used (Table 1). We applied the diffusion gradients in 20 noncollinear directions with *b* = 400 s/mm^2^ in addition to *b* = 0 s/mm^2^ **(**Figure 5 **)**. Images were obtained without respiratory triggering (given pelvic location of kidney allografts respiration is not an issue). We used selective fat suppression with spectral adiabatic inversion recovery. Standard shimming was performed through the pre‐imaging with the Siemens' diffusion sequence. For all participants, a parallel imaging acceleration factor of 2 and a simultaneous multi‐slice factor of 2 were used. Our protocol parameters were aligned with the published consensus‐based technical parameters to perform DTI of the kidneys [21]. An additional DWI‐MRI was acquired in axial orientation with 8 b‐values (0, 10, 20, 50, 100, 200, 500, 1000 s/mm^2^) to assess the accuracy of DTI‐derived ADC values. No specific preparations, such as fasting or fluid intake restriction, were recommended before the MR examination. In controls, both kidneys were included.

**TABLE 1:** Acquisition parameters for Kidney MRI DTI and DWI.

MRI parameters	DTI parameters	DWI parameters
TR (msec)	2100	6400
TE (msec)	71	46
Slice thickness (mm)	3	4
Matrix size	128 X 128	134 X 134
Bandwidth (Hz/pixel)	1776	2332
Resolution (mm)	2.4 X 2.4	1.2 X 1.2
Diffusion weightings	2	8
B‐values (s/mm^2^)	0, 400	0,10,20,50 100 200, 5 001 000
No. of directions	20	4
No. of slices	18	35
No. of averages	2	1
Diffusion scheme	Bipolar	Monopolar
Echo spacing (msec)	0.63	0.54
EPI factor	128	108
Fat suppression	SPAIR	SPAIR
Orientation	Coronal	Axial
Parallel Imaging factor	2	2
SMS	2	2
Scan time (mins: secs)	1:54	4:11

Abbreviations: DTI, diffusion tensor imaging; DWI, diffusion‐weighted imaging; EPI, echo‐planar imaging; Hz, hertz; mm, millimetres; msec, milliseconds; SMS, simultaneous multi‐slice; SPAIR, spectral adiabatic inversion recovery; TE, echo time; TR, relaxation time.

### 2.4 MRI Post‐Processing

**FIGURE 6:**
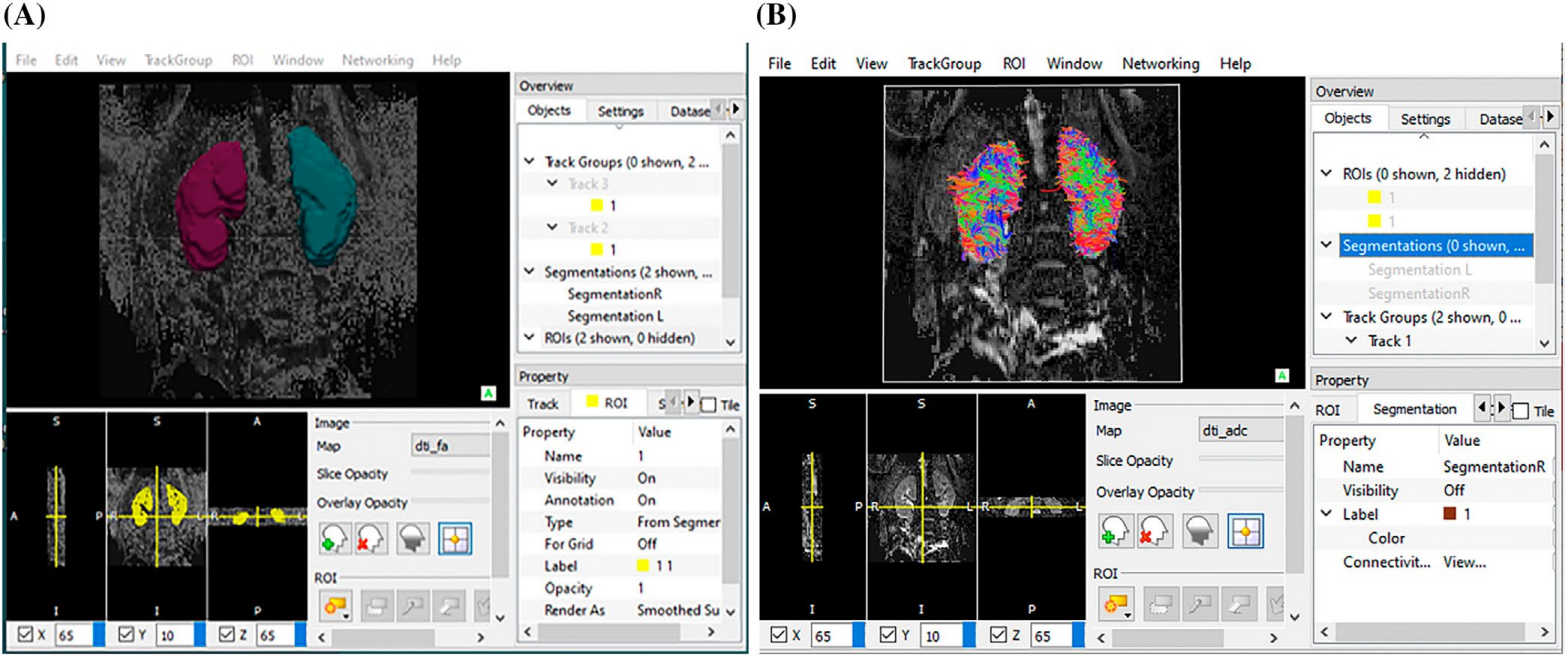
Representative snapshots from post‐processing pipeline of (A) whole kidney overlay with ROI mask and (B) whole kidney direction‐coded DTI map from a control subject with normal kidneys.

3D Slicer software (Brigham and Women's Hospital, Boston, MA) was used for manual segmentation of DTI kidney images [33]. The kidney cortex was segmented, avoiding areas of artifacts as well as avoiding inclusion of vessels and focal lesions. DTI maps were created using Diffusion Toolkit and TrackVis software (Martinos Center for Biomedical Imaging, Massachusetts General Hospital, Boston, MA) to create the diffusion metrics and tractography, respectively **(**Figure 6 **)**. The tensor was determined by the eigenvalues (λ_1_, λ_2_ and λ_3_).

Fractional anisotropy (FA) maps were calculated to depict the degree of diffusion anisotropy. Tracking was done using the tensor data and by generating kidney fiber tracks. Whole kidney tractography was performed with an FA threshold of 0.1 and an angle threshold of 55°. TrackVis interface allows for visualization of the fiber tracks in the sagittal, coronal, and axial planes (denominated as x, y, and z). When these planes are deselected, fiber tracks throughout the kidney can be visualized overlaid on a 2D image. This step allows for a complete visualization of the fibers on the kidney. The fibers oriented anterior‐to‐posterior are color‐coded as green, superior‐to‐inferior as blue, and left‐to‐right as red.

### 2.5 Statistics

Associations in DTI metrics (track length and FA) between kidney allografts and healthy controls were evaluated. In a sub‐analysis, we explored the association of DTI metrics among kidney allografts with histopathologic findings of rejection and CADI scoring [34, 35]. Patients were assigned to two groups according to allograft rejection: with and without rejection; and according to CADI score: CADI score 2+ (high) and CADI score < 2 (low). An additional sub‐analysis was performed to compare DTI metrics among kidney allografts that underwent “surveillance” versus “for‐cause” biopsy, given the difference in pretest probabilities for rejection in these two groups [36]. Normality was tested using the Shapiro–Wilk test. Variables with a normal distribution were compared using a two‐sample t‐test, while the Wilcoxon rank‐sum test was used for non‐normally distributed data. All analyses were performed with STATA (version 18; StataCorp LLC; College Station, Texas).

## 3 Results

**FIGURE 7:**
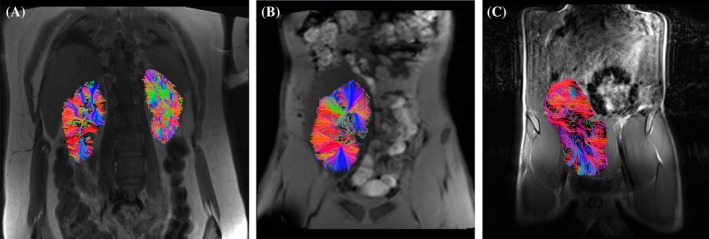
Representative images and DTI tracks generated from (A) control with native kidney versus (B) kidney transplant recipient with CADI < 2 and (C) kidney transplant recipient with CADI 2 + .

Fifty‐eight kidney transplant recipients were approached after screening for eligibility, and 26 were recruited (45% recruitment success rate). Fifteen kidney transplant recipients (4F/11M, median age = 16 years, IQR = 13–18 years, median kidney donor age = 31 years, IQR 23–36 years) (Table 2) were successfully enrolled in the MRI arm of the study and scanned with coronal DTI **(**Figure 7 **)** and axial DWI with satisfactory quality. The remaining 11 subjects were recruited but were unable to schedule their MR studies before their biopsies. In addition, 15 healthy children (9F/6M, median age = 15 years, IQR = 12–22 years) were recruited as controls out of 43 approached.

**TABLE 2:** IMPAKT participants' characteristics.

Characteristic^s^	Kidney transplant recipients (*n* = 15)	healthy controls (*n* = 15)
Age (years): median (IQR)	16 (13–18)	15 (12–22)
Males: *n* (%)	11 (73.3)	6 (40)
Donor status deceased: *n* (%)	9 (60)	n/a
Donor age (years): mean ± SD	31.27 ± 7.31	n/a
Underlying etiology of ESKD: *n* (%)	CAKUT: 10 (66.7) Genetic kidney disease: 2 (13.3) Glomerular: 2 (13.3) Other: 1 (6.7)	n/a
Surveillance vs. “for‐cause” biopsy *n* (%)	Surveillance biopsy: 6 (40) For‐cause biopsy: 9 (60)	n/a
Time after transplant (months): median (IQR)	17 (6–77)	n/a
Serum creatinine: mean ± SD	1.28 ± 0.53	0.65 ± 0.22
Proteinuria at baseline^b^: *n* (%)	Negative = 11 (73.33) Trace = 2 (13.33) 1+ = 1 (6.67) 2+ = 1 (6.67)	n/a

Abbreviations: CAKUT, congenital anomalies of the kidney and urinary tract; ESKD, end‐stage kidney disease; IQR, Interquartile range; n/a, not applicable; SD: standard deviation.

^a^ Mean values are reported for variables with normal distributions, while median values are used for variables with skewed distributions.

^b^ Proteinuria at baseline is defined as the level measured within 1 month before or after the MRI visit.

At the time of biopsy, 10 out of 15 kidney transplant recipients (66.7%) were receiving maintenance triple immunosuppressive therapy consisting of tacrolimus, steroids, and mycophenolate. Four subjects were on mono or dual therapy with the same agents and only one subject was on a combination of tacrolimus, steroids, and leflunomide. None of the kidney allografts included in the study experienced vascular complications at the time of transplant and no major post‐transplant complications. Five patients had prior biopsies before the IMPAKT study biopsy, one surveillance and four due to elevated or fluctuating creatinine. No significant differences were recorded among both (left and right) kidneys in controls. A significant difference was observed between kidney allografts (mean 19.36 mm SD ± 5.21) and control kidneys for track length (12.80 ± 3.34 mm, *p* < 0.001) and FA values: median 0.25 IQR (0.25–0.28) vs. 0.28 (0.27–0.33), *p* = 0.003 (Table 3). There were no statistical differences among kidney allografts in FA when compared by rejection status: median: 0.25, IQR (0.18–0.27) with rejection (*n* = 5) vs. 0.26 (0.25–0.28), without rejection [*n* = 10], *p* = 0.27. Similarly, FA values did not differ significantly between CADI score groups: 0.25 (0.25–0.28) CADI 2+ (*n* = 11) vs. 0.26 (0.24–0.34) CADI < 2 (*n* = 4), *p* = 0.46. When analyzing track length, no significant differences were found when comparing by rejection status and CADI score (Table 4). Mean ADC obtained by the 8 b‐value acquisition was 2428 × 10^−6^ mm^2^/s in transplanted kidneys vs. 2656 × 10^−6^ mm^2^/s in controls, with no statistically significant difference (*p* = 0.09). Mean ADC from DTI was 1484 x10^−6^ mm^2^/s in transplanted kidneys vs. 1361 x 10^−6^ mm^2^/s in controls, with no statistically significant difference (*p* = 0.11) (Table 3). Kidney function characteristics of controls and transplant recipients are reported in Table S1.

**TABLE 3:** Kidney DTI parameters in kidney transplant recipients and healthy controls.

DTI parameter^a^	Kidney allografts (*n* = 15)	Healthy controls (*n*=15^c^)	*p*‐value
ADC^b^ (mm^2^/s): mean ± SD	2428 × 10^−6^ ± 465 × 10^−6^	2656 × 10^−6^ ± 369 × 10^−6^	0.09
FA: median (IQR)	0.25 (0.25–0.28)	0.28 (0.27–0.33)	0.003
Track length (mm): mean ± SD	19.36 ± 5.21	12.80 ± 3.34	< 0.001

Abbreviations: ADC, apparent diffusion coefficient; DTI, diffusion tensor imaging; FA, fractional anisotropy; SD: standard deviation.

^a^ Mean values are reported for variables with normal distributions, while median values are used for variables with skewed distributions.

^b^ In two transplant recipients, the kidney could not be visualized, leading to two missing ADC values. As a result, there are 13 ADC values available for kidney transplant recipients.

^c^ There were 15 healthy controls included in the study, with data collected for each kidney, resulting in a total of 30 kidney units available for analysis.

**TABLE 4:** Kidney DTI parameters among kidney transplant recipients according to rejection and CADI scoring.

DTI parameter^a^	Kidney allograft subgroups			
	+ Rejection (*n* = 5)	Without Rejection (*n* = 10)	CADI 2+ (*n* = 11)	CADI < 2 (*n* = 4)
ADC^b^ (mm^2^/s): median (IQR)	2279 (2255–2466) × 10^−6^	2701 (2279–2839) × 10^−6^	2520 (2255–2683) × 10^−6^	2739 (1984–2882) × 10^−6^
*p*‐value	0.24		0.67	
FA: median (IQR)	0.25 (0.18–0.27)	0.26 (0.25–0.28)	0.25 (0.25–0.28)	0.26 (0.24–0.34)
*p*‐value	0.27		0.46	
Track Length (mm): median (IQR)	21.36 (19.75–22.56)	17.67 (14.97–19.15)	17.67 (14.97–21.36)	20.89 (17.46–28.29)
*p*‐value	0.14		0.12	

Abbreviations: ADC, apparent diffusion coefficient; CADI, chronic allograft damage index; DTI, diffusion tensor imaging; FA, fractional anisotropy; SD, standard deviation.

^a^ Mean values are reported for variables with normal distributions, while median values are used for variables with skewed distributions.

^b^ In two transplant recipients, the kidney could not be visualized, leading to two missing ADC values. As a result, there are 13 ADC values available for kidney transplant recipients.

ADC from the 8‐b‐value acquisition (median 2466 in surveillance vs. 2672.5 in for‐cause biopsies, *p* = 0.44), FA (0.28 vs. 0.25, *p* = 0.09), and track length (18.7 vs. 19.02, *p*‐value: 0.63) did not significantly differ between kidney allografts that underwent “surveillance” and those that underwent “for‐cause” biopsy.

## 4 Discussion

In this IMPAKT pilot study, we determined that prospective MR imaging performed prior to clinical biopsies among pediatric kidney transplant recipients is feasible, with a success rate of 100% of high‐quality images obtained for analysis in 15 kidney transplant recipients. Most patients were on standard triple immunosuppression with tacrolimus, steroids, and mycophenolate, a regimen comparable to adult protocols. In both populations, tacrolimus and mycophenolate are frequently used in combination as a maintenance therapy, with steroids included in patients with increased immunologic risk or concurrent autoimmune diseases [37].

When comparing DTI parameters between pediatric kidney allografts and healthy controls, we found a significant difference in the FA values, with lower values in kidney allografts (0.26 ± 0.06) than in control kidneys (0.32 ± 0.09). Low FA values refer to similar diffusion along all directions, whereas high FA implies preferential diffusivity occurring along one dominant direction. Healthy kidneys have a well‐defined structure with radial orientation of tubules, collecting ducts, and blood vessels, which makes diffusion properties in the kidney demonstrate obvious anisotropy [16, 17], exhibiting higher FA values. Our findings suggest that kidney allografts result in loss of coherent normal orientations, resulting in multi‐directional water diffusion and, hence, relatively low FA values. Our FA pediatric control values are congruent to those previously reported from our group, adding to the reference values in otherwise healthy children with no kidney disease [16].

Mean track length was significantly higher in kidney transplant recipients than healthy controls kidneys, which we believe is because the transplanted kidneys were adult donor kidneys (median donor age: 31 years (IQR 23:36 years) vs. healthy controls median age 15 years (IQR 12:22 years)). Our FA values of kidney transplant recipients (adult donor) match closely with previously reported healthy adult kidneys [38]. Shorter track lengths would otherwise be expected in kidneys with structural damage. For example, mean track length was significantly shorter in children and young adults with autosomal recessive polycystic kidney disease (ARPKD) than in healthy controls, despite having much higher track volumes [16]. In ARPKD, these differences are explained by the presence of cortical cysts that disrupt the normal tubular unidirectionality [16].

Among the subgroups of kidney transplants, we did not observe significant differences between allografts with and without rejection, nor between those with a CADI score below or above 2, likely due to a small sample size. Nevertheless, there is a potential for DTI quantitative metrics in detecting allograft dysfunction, as in a prospective pilot study by Li et al. on 16 pediatric allograft recipients, the authors found that in the localized post‐biopsy area, the *medullary* FA values inversely correlated with the 2013 single Banff criteria of tubulitis, interstitial inflammation, and tubular atrophy, characteristics of cellular allograft rejection and chronicity [35]. This finding might differ from our results due to two main reasons: one, that the histopathologic criterion was analyzed using the *single* 2013 Banff criteria, while we are using the 2017 *combined* Banff criteria to determine rejection and chronicity. The lack of consistent and standard ways of reporting Banff scores in research is a well‐known limitation of Banff. Secondly, Li et al. identified the location of the biopsy sample at the time of biopsy, which likely increased the precision of having an MR image and its *medullary* FA values correlate with their histological findings; we measured only cortical FA values. In this, we would argue that the value of a “whole” kidney marker, not limited to the biopsy area, challenges the inherent limitations of a biopsy sample, and therefore likely our *cortical* FA values, being more like Li et al.'s *cortical* FA values, are more representative of the current clinical practice. In another prospective assessment of 22 adult kidney transplant recipients, the authors observed a statistically significant difference in FA values between rejected transplanted kidney allografts and stable transplanted kidney allografts [39]. Similar to our study, in a comparison of 15 kidney transplanted patients with allograft dysfunction and 14 healthy volunteers, the authors reported that the ADC and FA were lower in transplanted than in healthy kidneys [40]. Given the variability found in the literature with single quantifiable MR parameters, next we will focus on building a combined radiomics model to better characterize our cohort, as previously done in diabetic kidney disease [41].

Diffusion MRI metrics including ADC, FA, and track length were similar between kidney allografts that underwent “surveillance” and “for‐cause” biopsy. The lack of differences may be due to the small sample size. To our knowledge, few studies have directly compared these metrics between surveillance and for‐cause groups. Although lower ADC values have been associated with impaired graft function, diffusion MRI has had mixed results in distinguishing between causes such as acute rejection [42]. FA has also been linked to graft fibrosis early after transplantation, but without specific comparisons between “surveillance” and “for‐cause” biopsies [43].

For MR‐DTI of the kidney to be used for clinical decision making, the technique needs to be consistent and diffusion metrics need to be reliably obtained. In our study, DTI metrics (track length, ADC and FA) were obtained from kidneys on every subject, and significant differences in track length and FA were observed between native and transplanted kidneys. The superimposed DTI fiber tractography over clinical MR images allows clinicians to have a better understanding of the additional information provided using DTI, which cannot be obtained with conventional MR images.

Our study had limitations, mainly those of the relatively small sample size and the unequal gender distribution. However, our main objective was to test the feasibility pilot of DTI on patients with kidney transplants, and even with a relatively small sample size, we were able to show some statistically significant differences. One potential limitation is that the control kidneys were matched by the age of the transplant recipient rather than kidney donor age. This may create some bias in that older kidneys may have altered microstructural changes that could potentially impact DTI parameters. Next, the underlying pathologic conditions that led to impaired kidney function were heterogeneous. Thus, the potential for DTI and DWI to differentiate between pathologic conditions (e.g., acute rejection, acute tubular necrosis) cannot be derived from our results. Differences were not found when comparing DTI metrics by rejection status and CADI score among kidney transplant allografts; however, the low post hoc power of our analysis (10%–30%) suggests that these comparisons may require a larger sample size to detect potential differences. Furthermore, we did not standardize the hydration state of our patients. Chandarana et al. showed that the hydration state may have an influence on measured kidney FA values [44]. By performing two DTI acquisitions in healthy patients who first refrained from fluid intake for 6 h and then underwent water loading, the authors observed a variation from 10% to 15% in FA values. Most of our transplant recipients got an early morning MRI scan before their biopsy and were recommended to be NPO (nothing by mouth) after midnight. The healthy controls were recommended to be hydrated normally and did not get any specific recommendations for fluid intake. Further studies with larger numbers of participants are warranted.

## 5 Conclusion

DTI of the kidney shows significantly different FA and track length values between transplanted and control kidneys. These results demonstrate the feasibility of identifying potential quantitative DTI metrics as a screening non‐invasive biomarker for evaluating pediatric kidney allograft health.

## Ethics Statement

All procedures performed in studies involving human participants were in accordance with the ethical standards of the institutional and/or national research committee and with the 1964 Helsinki Declaration and its later amendments or comparable ethical standards. The institutional review board at our hospital approved this prospective Health Insurance Portability and Accountability Act (HIPAA)‐compliant study. A statement has been included in the methods section of the manuscript.

## Conflicts of Interest

The authors declare no conflicts of interest.

## Supporting information


**Table S1.** Kidney function comparison among IMPAKT participants.

## Data Availability

Data can be made available on a reasonable request.
