# Supplementary material for: Diffusion Tensor Imaging (DTI) as a Non‐Invasive Tool for Assessing Pediatric Kidney Transplants: A Feasibility Study
Source: Pediatr Transplant. 2025 Jun 11;29(5):e70119. doi: 10.1111/petr.70119 (PMC12159281; doi:10.1111/petr.70119)
Supplement: Supplementary file 1 — Table S1. Kidney function comparison among IMPAKT participants. [file PETR-29-e70119-s001.docx]

**Supplement Table 1:** Kidney function comparison among IMPAKT participants

| IMPAKT participants^a^ | eGFR (mL/min/1.73m^2^)  mean ± SD | *p* value |
| --- | --- | --- |
| Kidney allografts (n=15): mean ± SD | 60.27 ± 17.43 | 0.001 |
| Healthy controls (n=15^b^): mean ± SD | 110.9 ± 11.40 |  |
| + Rejection (n=5): mean ± SD | 53.98 (19.06) | 0.34 |
| Without rejection (n=10): mean ± SD | 63.41 (16.67) |  |
| CADI 2+ (n = 11): mean ± SD | 55.40 ± 17.09 | 0.07 |
| CADI <2 (n = 4): mean ± SD | 73.64 ± 10.86 |  |

CADI: chronic allograft damage index. eGFR: estimated glomerular filtration rate used U25eGFR (ref). SD: standard deviation.

^a^Mean values are reported for variables with normal distributions, while median values are used for variables with skewed distributions.

^b^There were 15 healthy controls included in the study, with data collected for each kidney, resulting in a total of 30 kidney units available for analysis.
